# High SARS-CoV-2 seroprevalence among street adolescents in Lomé, Togo, 2021

**DOI:** 10.1186/s12879-023-08167-2

**Published:** 2023-04-03

**Authors:** Arnold Junior Sadio, Valentine Marie Ferré, Rodion Yao Konu, Anoumou Claver Dagnra, Diane Descamps, Didier Koumavi Ekouevi, Charlotte Charpentier

**Affiliations:** 1 Faculty of Health Sciences, Department of Public Health, University of Lomé, Center for Training and Research in Public Health, Lomé, Togo; 2 African Center for Research in Epidemiology and Public Health (CARESP), Lomé, Togo; 3 Research Institute for Sustainable Development (IRD), University of Bordeaux, National Institute for Health and Medical Research (INSERM), Bordeaux Population Health Centre, UMR 1219, Bordeaux, France; 4 Paris Cité University and Sorbonne Paris Nord University, IAME, Inserm, Paris, F-75018 France; 5 Virology Unit, AP-HP, Hôpital Bichat-Claude Bernard, Paris, F-75018 France; 6 Laboratory of Molecular Biology and Immunology, University of Lomé, Lomé, Togo

**Keywords:** SARS-CoV-2 seroprevalence, Street adolescents, Togo

## Abstract

**Background:**

There is almost no data on the circulation of SARS-CoV-2 among street adolescents. We conducted a study to document the immunization status of street adolescents in Togo against different variants of SARS-CoV-2.

**Methods:**

A cross-sectional study was carried out in 2021 in Lomé, the city with the highest number of COVID 19 cases in Togo (60%). Adolescents aged 13- and 19 years old living on the street were eligible for inclusion. A standardized questionnaire was administered face-to-face to adolescents. A sample of blood was taken and aliquots of plasma were transported to the virology laboratory of the *Hôpital Bichat-Claude Bernard (Paris, France)*. SARS-CoV-2 anti-S and anti-N IgG were measured using chemiluminescent microparticle immunoassay. A quantitative miniaturized and parallel-arranged ELISA assay was used to detect IgG antibodies specifically directed against the different SARS-CoV-2 Variants of Concern (VOC).

**Results:**

A total of 299 street adolescents (5.2% female), median age 15 years, interquartile range (14-17 years), were included in this study. The prevalence of SARS-CoV-2 infection was 63.5% (95%CI: 57.8–69.0). Specific-IgG against the ancestral Wuhan strain was developed by 92.0% of subjects. The proportion of patients being immunized against each VOC was 86.8%, 51.1%, 56.3%, 60.0, and 30.5% for the Alpha, Beta, Gamma, Delta, and Omicron VOCs, respectively.

**Conclusion:**

This study showed a very high prevalence with approximately 2/3 of Togolese street adolescents having antibodies to SARS-CoV-2 due to a previous infection. These results confirm an under-reporting of COVID-19 cases in Togo, questioning the hypothesis of low virus circulation in Togo and even in Africa.

## Introduction

The true magnitude of the SARS-CoV-2 epidemic is unknown in sub-Saharan Africa. According to existing data, only 1.7% of Covid-19 cases have been reported in Africa out of more than 517 million cases worldwide [1]. One way to estimate the true extent of the epidemic is to conduct seroprevalence surveys [2]. The last study conducted in Togo in 2021, in twelve health districts, with the inclusion of more than 7000 people, reported a seroprevalence of 65.5% [3]. These studies are essentially household surveys, which are costly and time-consuming and therefore difficult to repeat over time. Epidemiological surveillance surveys can also be carried out in specific populations, as is the case in France where a study was carried out among homeless people and showed a circulation of the virus among this population after the first epidemic wave [4]. Homeless people are particularly vulnerable to SARS-CoV-2 infection. Indeed, several risk factors for SARS-CoV-2 infection are found in these populations, including promiscuity, multiple residence in often poorly ventilated dwellings, and frequent contact with many people in community support services. In addition, they are at increased risk for severe COVID-19, being exposed to a high prevalence of comorbidities [5–7].

In Africa, there are populations that live in conditions as precarious as the homeless and are just as vulnerable: street adolescents. This is a very mobile population that is often found in city markets, border crossings and roadside pay stations. This population of street adolescents was on the almost excluded of all risk mitigation measures taken by governments. In 2021, we conducted a study among street adolescents to assess the feasibility of HIV self-testing. As this study was conducted in the midst of the COVID-19 pandemic, and considering the lack of epidemiological data in the literature on SARS-CoV-2 infection in this population, we conducted an ancillary study to document the immunization status of street adolescents in Togo against different variants of SARS-CoV-2.

## Methods

### Study design and setting

This study was part of a survey aimed at describing the acceptability and feasibility of HIV self-testing among street adolescents in Lomé (the capital city of Togo). This was a cross-sectional study conducted from june 26th to july 3rd, 2021 after the second wave of pandemics.

Togo is a West African country covering a 56,800 km² area with an average density of 145 inhabitants per square kilometer [8]. The population was 8.08 million in 2019, of which 50.2% were women [9]. Most of the population is under 25 years of age (60%), and lives in rural areas (62%) [9]. Togo’s health system has a pyramidal structure with three levels: central, intermediate and peripheral. Each level has administrative and health care delivery components. Lomé is the largest urban center in the country and at the time of the survey, the city alone accounted for more than 60% of reported COVID-19 cases in Togo [10].

### Study population and sample size

All adolescents aged between 13- and 19-years old living in the street were eligible for inclusion following informed consent. After listing all the places where street adolescents gather in Lomé, a team of investigators went to each site accompanied by local NGOs working with the street child population. After explaining the study to the adolescents, those who agreed to participate were asked to sign a consent form if they were over 18 years old. Adolescents under the age of 18 were asked to give their assent to participate in the study, and then a member of the NGO involved in the care of street adolescents was asked to sign a consent form.

### Data collection

A standardized questionnaire was administered face-to-face to adolescents. It included socio-demographic characteristics, sexual practices, and history of HIV testing. Then, a sample of 04 ml of venous blood was taken.

### Laboratory procedures

Aliquots of plasma were taken to the laboratory of molecular biology and immunology of the University of Lomé (Lomé, Togo) and transported frozen to the virology laboratory of the *Hôpital Bichat-Claude Bernard (Paris, France)*, for the search for anti-SARS-CoV-2 antibodies.

SARS-CoV-2 anti-S and anti-N IgG were measured using the automated Abbott SARS-CoV-2 IgG kit (chemiluminescent microparticle immunoassay, CLIA) (Abbott, IL, USA) using the Alinity platform according to the manufacturer’s instructions. In addition, the CoViDiag kit (SirYus CoViDiag+, Innobiochips®, Loos, France), a quantitative miniaturized and parallel-arranged ELISA assay [11], was used to detect IgG antibodies specifically directed against the different SARS-CoV-2 Variants Of Concern (VOC) including BA.1 Omicron sublineage with a threshold at 18 BAU/mL. CoViDiag microtitration plates wells are coated with 4 different SARS-CoV-2 antigens and 4 RBD domains from different VOC separately. They are dedicated to bind specific antibodies in the tested samples and therefore deliver different responses in one single assay.

### Case definition

A sample was considered positive if any of the following conditions were met: (i) the presence of anti-protein S antibodies; (ii) the presence of anti-N antibodies; or (iii) a result in the greyzone of the assays. An humoral response was defined as having a positive titer against the VOC using the COVIDIAG assay.

### Statistical analysis

We performed descriptive statistics, and the results were presented using frequency tabulations and percentages for categorical variables. Quantitative variables were presented as medians with their interquartile range (IQR). Kruskal-Wallis rank sum test or Wilcoxon test were used for comparison when appropriate. Prevalences of SARS-CoV-2 antibodies were estimated with their 95% confidence interval (95%CI).

## Results

A total of 299 street adolescents, median age 15 years, interquartile range (IQR) (14-17 years), of which 5.2% (n = 16) were female were included in this study. Among these, 246 out of 299 (82.3%) are of Togolese nationality (Table 1).

Anti-S IgG serology was positive for 190 of the 299 tested samples leading to a prevalence of SARS-CoV-2 infection of 63.5% (CI 95%: 57.8–69.0). Anti-N IgG antibodies, marker of a more recent infection, were positive for 125 samples (41.8%; CI 95%: 36.2–47.6) (Table 1).

**Table 1:** Seroprevalence of SARS-CoV-2 antibodies by sociodemographic characteristics

	N	Anti-S antibody					Anti-N antibody			
		n	%		95%CI		n	%		95%CI
**Overall**	299	190	63.5		57.8–69.0		125	41.8		36.2–47.6
**Age (years)**										
**Median [IQR]**	15	[14–17]								
10–14	105	63	60.0		50.0–69.3		40	38.1		28.9–48.1
15–19	194	127	65.5		58.3–72.0		85	43.8		36.8–51.1
**Sex**										
Female	16	9	56.2		30.6–79.2		4	25.0		8.3–52.6
Male	283	181	64.0		58.0–69.5		121	42.8		36.9–48.8
**Nationality**										
Other	53	36	67.9		53.6–79.7		22	41.5		28.4–55.8
Togolese	246	154	62.6		56.2–68.6		103	41.9		35.7–48.3
**Education level**										
No academic	23	13	56.5		34.9–76.1		13	56.5		34.9–76.1
Primary	121	75	62.0		52.6–70.5		40	33.1		24.9–42.3
Secondary	121	81	66.9		57.7–75.1		55	45.5		36.5–54.7
Superior	34	21	61.8		43.6–77.3		17	50.0		34.1–65.9
**HIV serology**										
Negative	296	189	63.9		58.1–69.3		125	42.2		36.6–48.1
Positive	3	1	33.3		1.8–87.5		0	0.0		0.0–69.0

**Fig. 1:**
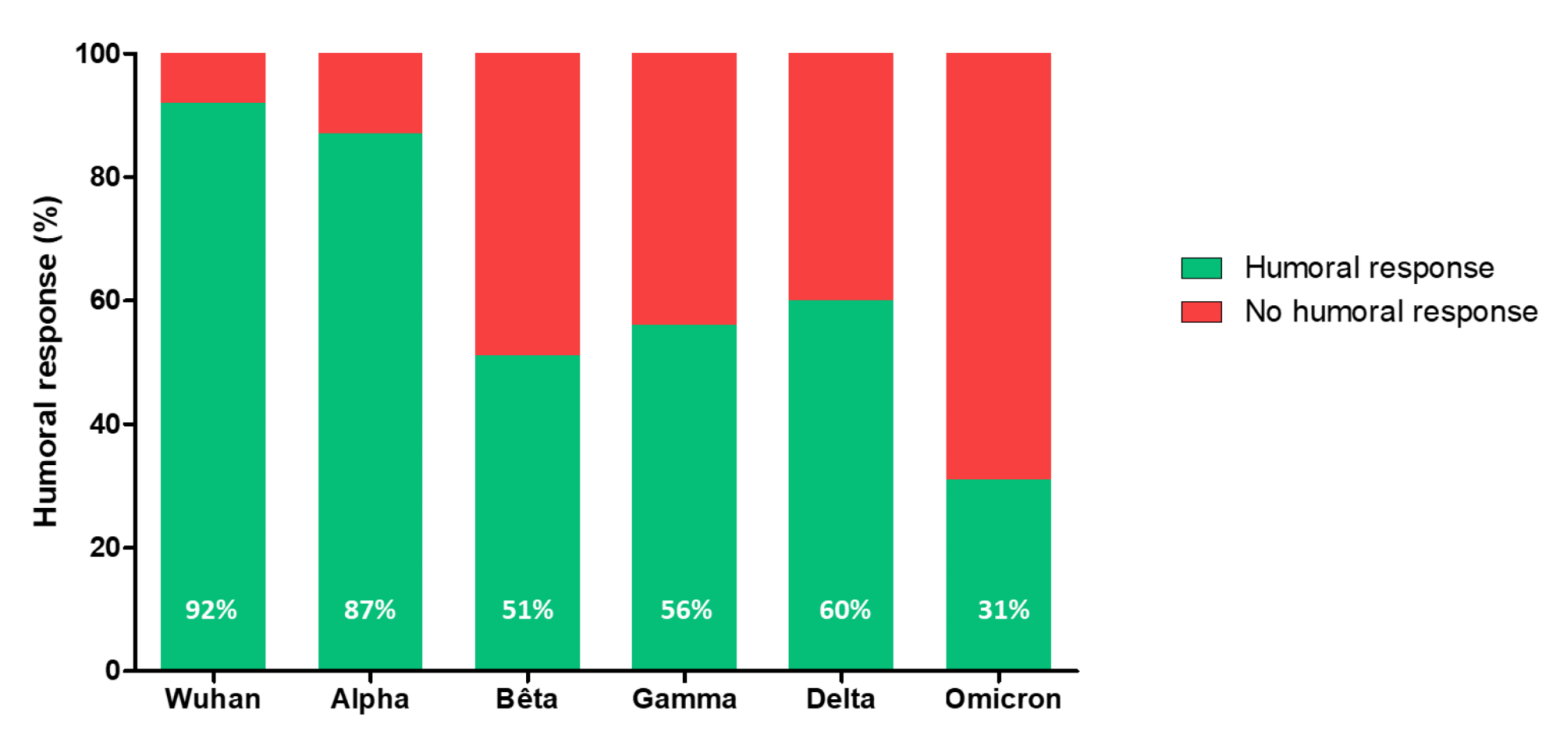
Proportion of population harbouring humoral response against the SARS-CoV-2 Spike RBD of the different strains studied among the participants tested positive for anti-S SARS-CoV-2 IgG in ELISA (n = 190)

VOC-specific anti-S IgG titers were assessed for the 190 samples with positive anti-S IgG. 175/190 (92.0%) subjects developed specific-IgG against the ancestral Wuhan strain. The 8% of subjects showing no anti-S IgG against the ancestral Wuhan strain had anti-S IgG titers ≤ 18 BAU/mL, which is the LOQ of the multiplex ELISA assay. The proportion of patients with presence of anti-S IgG against each VOC was 86.8% (n = 165/190), 51.1% (n = 97/190), 56.3% (n = 107/190), 60.0% (n = 114/190), and 30.5% (n = 58/190) for the Alpha, Beta, Gamma, Delta and Omicron VOCs, respectively (Fig. 1). The proportion of the population harbouring a humoral response against the ancestral strain and the Alpha VOC were significantly higher than against all other VOCs (p < 0.0001 for Beta, Gamma, Delta and Omicron VOCs).

**Fig. 2:**
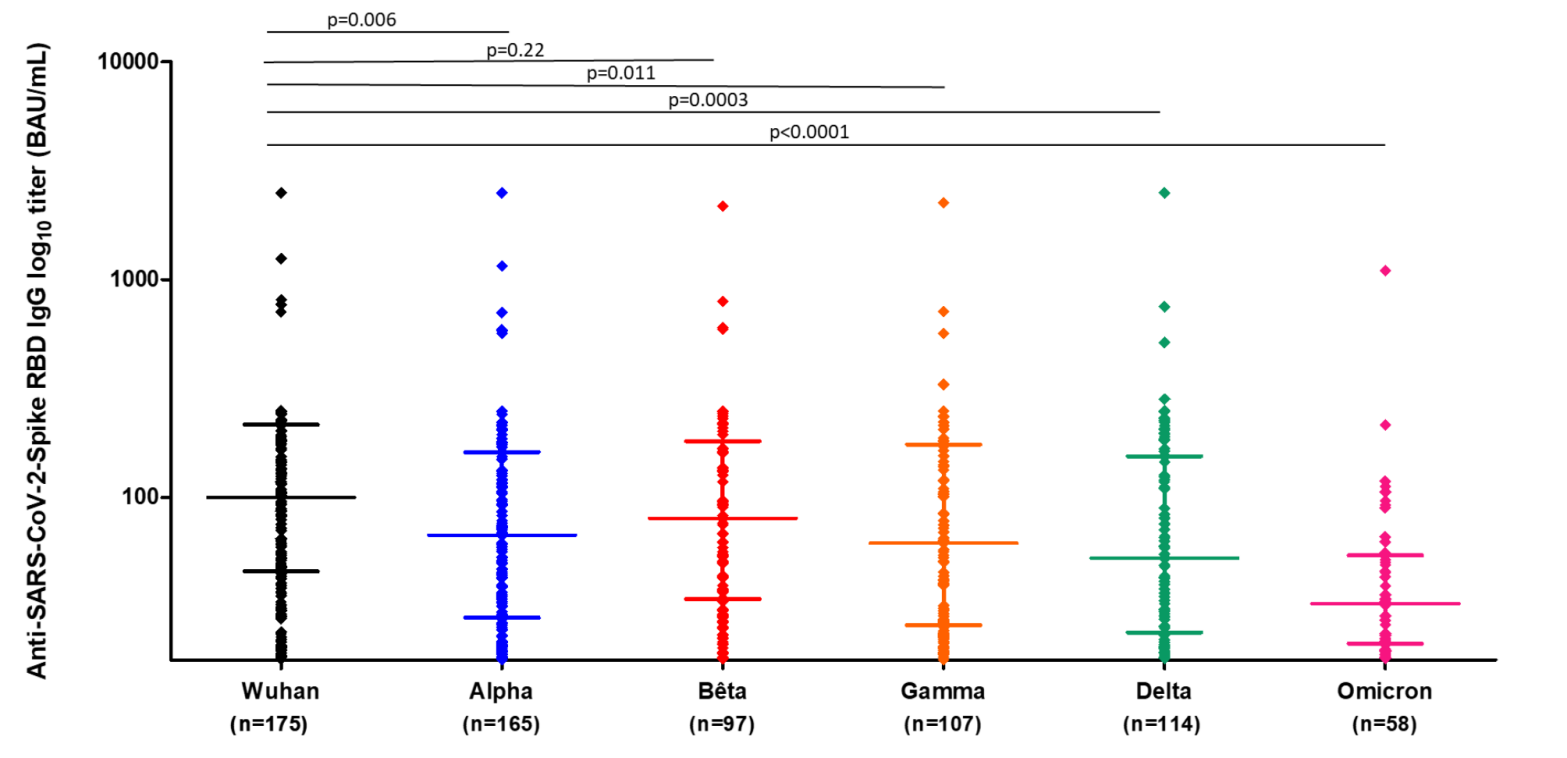
Titers of IgG antibodies anti-SARS-CoV-2 Spike RBD of different variants using the COVIDIAG multiplex ELISA assay measured in the population harbouring a positive SARS-CoV-2 anti-S IgG serology (n = 190)

Among subjects who developed an immunity against one or several VOCs, the median titers were 68 BAU/mL (IQR = 28–162), 81 (IQR = 34–182), 62 (IQR = 26–175), 53 (IQR = 24–155) and 33 (IQR = 21–54) for the Alpha, Beta, Gamma, Delta and Omicron VOCs, respectively (Fig. 2).

Among participants who showed an humoral response, Anti-S IgG median titers were significantly lower against Delta and Omicron variants compared to the ancestral Wuhan strain (p = 0.0003 and p < 0.0001, respectively). Regarding a potential humoral response against the Omicron variant not yet encountered by this population at the time of sampling, anti-S1 RBD IgG median titers were significantly lower against this future new VOC at that time compared to all the variants studied (p < 0.0001, p < 0.0001, p = 0.0002 and p = 0.0023 for Alpha, Beta, Gamma and Delta VOCs, respectively) (Fig. 2).

## Discussion

A seroprevalence study was conducted in a Togolese vulnerable population at high risk of infection because of non-compliance with government-mandated barrier measures to mitigate the risk of SARS-CoV-2 infection. A total of 2 out of 3 adolescents had anti-S antibodies demonstrating significant virus circulation as of June 2021. The results observed are comparable to those observed in the Togolese general population in April 2021 (65.5%) [3], and prove an under-reporting of SARS-CoV-2 infection in the country. Indeed, as of July 30, 2021, less than 40,000 cases (0.5% of the Togolese population) of SARS-CoV-2 infections were officially reported in Togo [10].

This study, which is the first seroprevalence study among the street’s adolescents, also documented recent infections with the identification of Anti-N IgG antibodies, which is not the case in the majority of SARS-CoV-2 seroprevalence surveys that document only antibodies targeting the Spike protein. The results show that 42% of street adolescents had a recent SARS-CoV-2 infection. Differential reactivity of S- and N-specific antibodies can be used to help differentiate prior infection from vaccination in serological studies, particularly for vaccines that produce antibodies only to the S protein [12–14]. The presence of isolated anti-S antibodies can also result from a past COVID-19 infection, after the disappearance of the anti-N antibodies, known to decrease more rapidly than the anti-S antibodies [15, 16]. However, for the present study, given the start date of vaccination in Togo and the target chosen at the beginning, it can be said that surveyed street adolescents were not vaccinated. Indeed, vaccination in Togo began on March 10, 2021, and was limited to health professionals and people aged 20 years and older at the end of the study.

Regarding the research of SARS-CoV-2 variants, this is not systematically carried out in Togo. It is only performed during epidemic peaks for surveillance purposes and to adjust the response measures against the pandemic. So, no naso-pharyngeal samples were available in this study, preventing to have a description of the circulating SARS-CoV-2 variants in this adolescent’s population. As of August 4, 2021, official genotyping data from samples collected in Togo reported 90% of Delta and 1.3% of Eta variant [17]. In December 2021 the same data reported 73.6% of Delta and 24.5% of Omicron [18]. The results of the COVIDIAG assay confirmed the capacity of immune escape of the Delta and Omicron VOCs with a lower proportion of patients immunized and if is the case in a lower level [19]. This assay was indeed proven to show a strong correlation between specific RBD antibodies titers and live viral neutralization ability of sera [19]. Thus, even if some serological cross-reactivity has been previously described with other serological assays on African samples [16], no such cross-reactivity is expected with the assays used in this study.

A major limitation of this study is that it was not initially designed for an SARS-CoV-2 investigation, which did not allow us to collect specific information about SARS-CoV-2 infection such as clinical manifestations of SARS-CoV-2 or history of screening or hospitalization related to COVID-19.

## Conclusion

This is the first seroprevalence study of SARS-CoV-2 conducted in sub-Saharan Africa among street adolescents in mid-2021. This study showed a very high prevalence with approximately 2/3 of Togolese street adolescents having antibodies to SARS-CoV-2 due to a previous infection. The seroprevalence data from this survey in a population with limited access to the health system and non-compliance with government measures confirm those observed in the general population. These results confirm an under-reporting of COVID-19 cases in Togo, questioning the hypothesis of a low circulation of the virus in Togo and even in Africa.

## Data Availability

The datasets used and/or analysed during the current study are available from the corresponding author upon reasonable request.
